# Dengue Virus Non-Structural Protein 5

**DOI:** 10.3390/v9040091

**Published:** 2017-04-24

**Authors:** Abbas El Sahili, Julien Lescar

**Affiliations:** School of Biological Sciences, Nanyang Technological University, Nanyang Institute for Structural Biology, Experimental Medicine Building, 59 Nanyang Drive, Singapore 636921, Singapore

**Keywords:** flavivirus, dengue virus, NS5 polymerase

## Abstract

The World Health Organization estimates that the yearly number of dengue cases averages 390 million. This mosquito-borne virus disease is endemic in over 100 countries and will probably continue spreading, given the observed trend in global warming. So far, there is no antiviral drug available against dengue, but a vaccine has been recently marketed. Dengue virus also serves as a prototype for the study of other pathogenic flaviviruses that are emerging, like West Nile virus and Zika virus. Upon viral entry into the host cell and fusion of the viral lipid membrane with the endosomal membrane, the viral RNA is released and expressed as a polyprotein, that is then matured into three structural and seven non-structural (NS) proteins. The envelope, membrane and capsid proteins form the viral particle while NS1-NS2A-NS2B-NS3-NS4A-NS4B and NS5 assemble inside a cellular replication complex, which is embedded in endoplasmic reticulum (ER)-derived vesicles. In addition to their roles in RNA replication within the infected cell, NS proteins help the virus escape the host innate immunity and reshape the host-cell inner structure. This review focuses on recent progress in characterizing the structure and functions of NS5, a protein responsible for the replication and capping of viral RNA that represents a promising drug target.

## 1. Introduction

Dengue virus is a mosquito-borne human pathogen affecting mostly inter-tropical regions, where 3.9 billion persons live, with nearly 100 million infections provoking clinical symptoms every year [1]. Transmission of the virus occurs through the bite of mosquitoes from the *Aedes* genus (mostly *Aedes aegypti* and *A. albopictus*). These mosquito vectors are commonly found in inter-tropical regions but now also in more temperate areas to due global warming, thus raising the possibility of further expansions of the areas affected by dengue. Most infections are asymptomatic or cause mild symptoms (fever, joint pain or rashes) lasting for a few days. However, in some severe cases, consequences can be more dramatic (dengue hemorrhagic fever, dengue shock syndrome) causing about 20,000 deaths per year. The dengue virus belongs to the flavivirus family along with West Nile virus (WNV), yellow fever virus and the recently emerging Zika virus (ZIKV). Dengue virus comprises four serotypes labelled DENV 1 to 4. The viral particle encapsulates a positive strand RNA of 11 kb that constitutes the viral genome, with 5’ and 3’ untranslated regions (UTR) and a 5’ cap. Upon infection, the RNA is translated into a single polypeptide chain embedded in endoplasmic reticulum (ER) membranes that is processed in ten proteins following proteolytic maturation by viral and host cell proteases. The envelope, the precursor-membrane and the capsid form the structural proteins encasing the viral RNA. NS1, NS2A, NS2B, NS3, NS4A, NS4B and NS5 are non-structural (NS) proteins expressed in the host cell that are essentially not incorporated in the viral particle. With the help of host proteins, NS proteins reshape the inner organization of the cell, mature the polyprotein, replicate the viral RNA and help the virus evade the immune system. Infection by dengue virus is followed by noticeable changes in the inner cell substructures, including formation of ER vesicles, double membrane particles formation and elongation of mitochondria [2]. The viral genome is translated as a long polypeptide chain in the rough ER, inserted in the membrane where it is matured. NS2A, NS2B, NS4A and NS4B are integral membrane proteins important for viral replication. As they do not possess any defined enzymatic activity; they are likely to act through protein–protein and protein–lipid interactions. The exact roles played by NS1, NS2A and NS4A are still elusive [3]. NS1 has different oligomerization states dependent on its glycosylation status [4,5,6]. In the infected cell, NS1 is bound to the ER vesicles membrane on the lumen side and helps anchoring the viral replication complex. However, it can also be secreted and is thought to help the virus evade the immune system. NS2A, also a membrane protein, has been very poorly characterized so far. Mis-cleavage between NS1 and NS2A can affect viral RNA replication, and mutations in the protein affects virion assembly [7]. NS4A acts as a scaffold for the replication complex and is proposed to induce membrane alterations [8,9,10,11]. It has been found to form oligomers and mutations in the protein affect virus replication but the underlying mechanism is still unknown. More information is available for NS2B-NS3, NS4B and NS5, indicating they are promising drug targets. NS3 is a 618-residue protein with two domains: the protease lies at the N-terminal end and uses the NS2B cytoplasmic loop as a cofactor [12] whilst the C-terminal domain possesses the helicase activity that presumably unwinds double-stranded RNA (dsRNA) [13,14] along with an ATPase activity involved in the capping of the newly synthetized genomic RNA. The helicase domain of the NS3 protein interacts with the cytoplasmic loop of NS4B [15]. 

**Figure 1:**
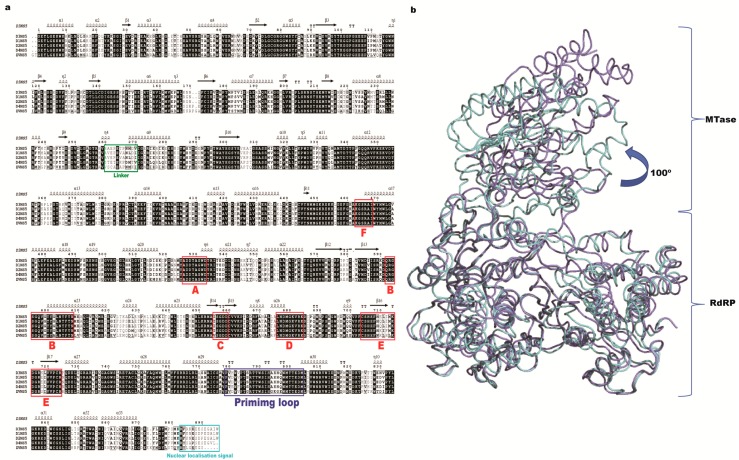
(**a**) Sequence alignment of dengue virus (DENV) NS5 proteins from the four serotypes. Sequence numbering is according to DENV2 NS5. Secondary structure assignment follows the DENV3 NS5 full-length protein structure (Protein Data Bank (PDB) access code 4V0Q). Specific sequence motifs (A–F) are labeled in red. The linker region is indicated in green. The recently characterized nuclear localization signal is colored in light blue and the priming loop in blue; (**b**) Superimposition between DENV 3 NS5 (4V0Q, light blue) and Zika virus (ZIKV) NS5 (5TFR, purple) full-length protein structure is represented as α-carbon traces. MTase: methyltransferase; RdRP: RNA-dependent RNA polymerase.

Here, we summarize recently published research on the NS5 protein, an important enzyme drug target. NS5 is the largest (102 kDa) and the most conserved protein (with ~70% sequence identity among the four serotypes, see Figure 1A) [16] expressed during infection by dengue virus. It carries two domains: a methyltransferase domain (MTase) at its N-terminal end and a RNA-dependent RNA polymerase (RdRp) at its C-terminus (Figure 1B). The architecture of the NS5 protein is well conserved across flaviviruses as confirmed by the recent crystal structure determination of the ZIKV NS5 protein [17,18,19,20]. This high level of structure conservation suggests that it is possible to design compounds targeting NS5 with broad activity against several flaviviruses. The MTase domain (residues 1–265) is responsible for capping the viral RNA and also has a putative guanylyl transferase and a N7 and 2’O ribose methylation activity. The RdRp domain replicates viral RNA. Both domains are connected by a linker of 5–6 residues (residues 266–271), which is an important determinant of the NS5 overall conformation and protein activity. The C-terminal domain (Figure 1B) (276–900) contains the RdRp enzymatic motifs. Because the RdRp activity is absent in the host cell, NS5 represents a promising antiviral target to design specific inhibitors with low toxicity. In addition to its role in replicating the viral genome, NS5 can also down-regulate the host immune interferon response, via its interaction with the signal transducer and activator of transcription 2 (STAT2) protein [21] or, as proposed recently, by modulating RNA splicing within the host cell [22].

## 2. Methyltransferase Domain

The MTase is responsible for capping the nascent genomic RNA sequentially using S-adenosylmethionine as the methyl donor, via sequential methylation on the N7 atom of the cap guanine and the 2’O atom of the ribose of the first strictly conserved adenine of the genome [23,24]. Viral capping resembles the 5’ capping of mRNA in the eukaryotic cell, prevents its degradation and enhances interaction with the ribosome for translation [25]. Defects in capping decrease viral multiplication and lead to attenuated viruses that are more sensitive to the innate immune response, as they induce higher interferon (IFN) signaling and antibody response [26]. Several pathogen recognition receptors like retinoic acid inducible gene I (RIG-I) or melanoma differentiation-associated protein 5 (MDA5) detect the presence of dsRNA or uncapped 5’ triphosphate RNA, that are produced during the flavivirus replication cycle [8]. The MTase domain capping activity helps the virus escape from these host cell sensors [27]. In summary, methylation of the viral RNA plays a major role allowing the virus to escape the immune response. Higher IFN sensitivity has been shown to result from defects in 2′-*O*-methylation on the penultimate A nucleotide of the genome, as it allows the viral RNA to escape recognition by IFN induced protein with tetratricopeptide repeats 1 (IFIT1) sensor proteins [28]

### 2.1. Overall Structure

**Figure 2:**
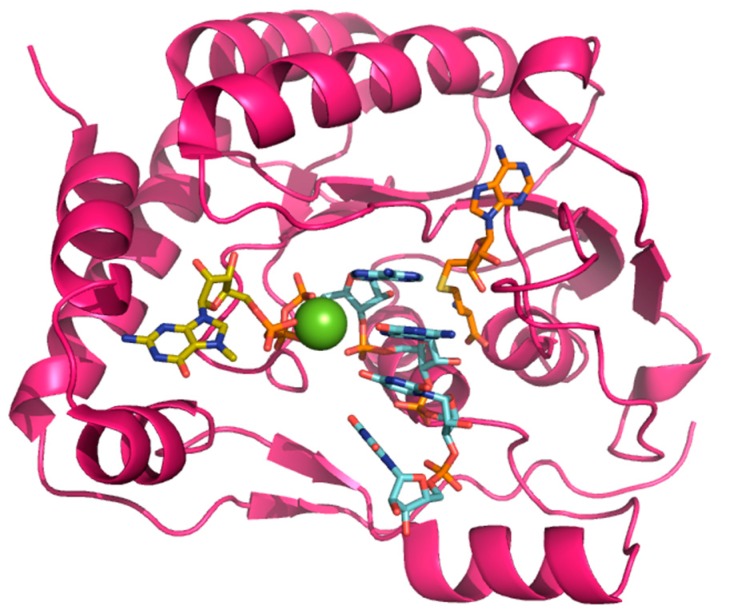
MTase domain structure bound to 7-methylguanosin (m7)-GpppAGUU. The MTase domain of DENV NS5 (PDB access code: 4V0Q) is shown as pink ribbons. The RNA with sequence 5’AGUU-3’ is shown as sticks and colored in light blue. The m7G and S-adenosyl-L-homocysteine (SAH) moieties are represented as sticks and colored in yellow and orange respectively.

Crystal structures of the MTase domain (residues 1–263) showed that it shares the characteristic α/β fold (Figure 2) as seen in the structures of DENV (Protein Data Base (PDB) access code: 3P97) [29], Japanese encephalitis virus (JEV, 4K6M) [30] or the recent structures of ZIKV (5KQR) [31] or WNV (3LKZ) [32]. Sequence identities between various flaviviruses range from 50 to 70%. The MTase domain can be subdivided into three subdomains. First, the C-terminal side possesses the characteristic MTase fold formed by a seven strands β-sheet surrounded by four α-helices. In several crystal structures, a S-adenosyl-L-homocysteine (SAH) molecule (the by-product of the methylation reaction) is found bound to this domain (Figure 2) [23]. On the N-terminal side, a helix-turn-helix motif, a β-strand and a α-helix form the second subdomain. From crystal structures bound to m7Gppp-RNA, this domain was proposed to coordinate the guanosine-5′-triphosphate (GTP) moiety of 7-methylguanosin-GTP (m7GTP) during the 2’-*O*-ribose methylation [23]. The third subdomain is found between the two previous ones and is composed of an α-helix and two β strands [33]. A magnesium ion is also present in the RNA-bound structure, coordinating the phosphates and help positioning the substrate for methyl transfer (Figure 2).

### 2.2. Guanylyltransferase Activity

Guanylyltransferase activity was proposed for NS5 based on in vitro experiments, using a triphosphorylated RNA strand in the presence of the NS5 and NS3 proteins and GTP [24]. The putative mechanism involves the 5’-triphophatase activity of the C-terminal helicase domain of NS3 to produce a diphosphate 5’ end to the RNA. In the case of Wesselsbron virus (a member of the flavivirus family, [34]), upon incubation with GTP, the MTase becomes covalently linked to guanosine monophosphate (GMP), via the residue K28 [34]. An enzyme-GMP covalent complex is also found in the case of the full length NS5 protein from WNV [24,35]. In flaviviruses, lysine 28 is not strictly conserved and can be substituted by an arginine and yet still retains the ability to covalently bind GMP. The GMP moiety would then be transferred onto the 5’ end of the RNA, to produce the GpppA cap moiety that is subsequently methylated on the N7 atom of the guanine base and the 2’O atom of the first adenine ribose. The guanylyltransferase activity of the MTase domain of NS5 is enhanced by the presence of NS3 [24,36], as shown using WNV proteins, probably through an intermolecular interaction involving the region surrounding the linker domain of NS5 and the subdomain 3 of the NS3 helicase domain. 

### 2.3. Methyltransferase Activity

The methyl group donor is an S-adenosylmethionine molecule bound inside a deep pocket within the MTase domain. A recent structural study of a ternary complex between the MTase (in the context of the full-length NS5 protein), the partially capped RNA (at the N7 position) and S-adenosyl-L-homocysteine (the by-product of the reaction) allowed the authors to propose a detailed mechanism for 2’-*O*-ribose methylation of genomic viral RNA [23]: The catalytic lysine 180 is the base that activates the 2’-OH group of the ribose to perform nucleophilic attack on the methyl group carbon atom of S-adenosyl methionine (SAM). The RNA is positioned through numerous interactions via its phosphate groups and a bound magnesium further stabilizes the complex. The binding of the guanine and the adenine is very specific. The guanine moiety of m7G is stabilized by stacking with the phenyl ring of a phenylalanine (F25) in the binding site. Interestingly, m7G is bound in the same pocket as the GMP prior to guanylyl transfer [23,24,34]. The adenosine is coordinated in a site close to the SAM-binding pocket [23]. Of the two different methylation reactions (one on the N7 of the guanine base and the other on the 2’-*O*-ribose of the adenine base [34,37]), 2’-*O*-methylation is proposed to occur after the *N7*-methylation [23,35]. Only one SAM-binding pocket is present per NS5 monomer, and as noted above, it is deeply buried in the protein, and release of the by-product requires displacement of the bound RNA. The only available structure of DENV protein with RNA was determined in presence of m7GpppA-capped RNA [23], capturing the complex during the second methylation step. As proposed by several authors, the question then arises as to whether one or more MTase subunits cooperate to transfer the guanylyl group, and to perform both methylation steps. Given the close proximity between the GMP/GTP pocket, (where the guanylyl transferase activity occurs and the m7G of the RNA is positioned during the 2’-*O*-ribose methylation) and the SAM pocket, it is likely that the three reactions involve dissociation followed by repositioning of the RNA for the following enzymatic step (Figure 2).

Recently, Gokhale et al. reported that the flavivirus genomic RNA is also internally methylated on adenosines [38]. Surprisingly, this RNA modification in the case of hepatitis C virus (HCV) is linked to down-regulation of HCV particle production. If cap addition and methylation has a clear functional role in RNA stabilization and in escaping cell sensors from the host innate immunity, the exact effects of these internal methylations on the flavivirus have not yet been described. However, the MTase domain of ZIKV was also found to methylate internal adenosine bases in its genome [39].

## 3. RdRp Domain

Upon infection, the positive sense viral RNA is released in the cytoplasm. NS5 protein first transcribes it as a negative sense strand before using the negative strand (in the context of a dsRNA intermediate) to synthesize a large excess of + RNA. The viral mRNA is then used to express the polyprotein by host cell ribosomes and also for encapsulation into new viral particles.

### 3.1. RdRp Domain Fold

**Figure 3:**
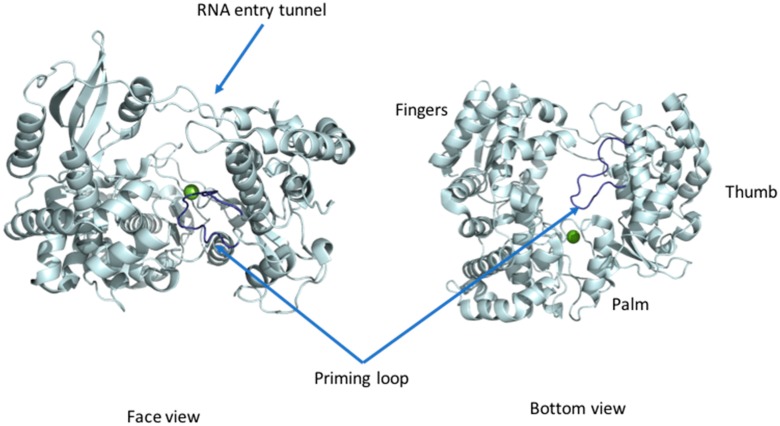
RdRP domain. The RdRp domain of DENV3 NS5 (4V0Q) is represented as ribbons and colored in light blue in front (left) and bottom view (right). The magnesium (green) ions are represented as spheres. The priming loop is colored in dark blue.

The first structure of the DENV RdRp domain (residues 270–900) published 10 years ago revealed a right-hand architecture found in many DNA and RNA polymerases, with a palm, a thumb and a fingers subdomain. Conserved motifs A–G each with a precise function are present in the flavivirus RdRp (Figure 1A and Figure 3): motif A contributes to the cation binding site [40], motif B helps in the sliding of the RNA in the RdRP tunnel [41,42], motif C comprises the GDD catalytic residues [40], motif D is proposed to help in the release of the PPi by-product [43], motif E houses the structural zinc cation [40], and motif F is proposed to help stabilize the nascent base pair [44]. Recent structures of the Zika virus NS5 RdRp domain confirmed the conservation of the RdRp fold, as was anticipated from the high amino-acid sequence conservation [18]. Together, these regions shape a flat structure traversed by three tunnels granting access to the template, to incoming ribonucleosides tri-phosphate (rNTPs) and allowing exit of the newly synthesized dsRNA product (Figure 3) [40]. The palm domain houses the active site with two aspartic acid residues (658 and 659 from polymerase motif C, using DENV2 numbering) chelating the two Mg metal ions and allowing formation of the phosphodiester bond. Moreover, a specific region called the “priming loop” that is a hallmark of RdRps able to catalyze polymerization “de novo” (e.g., in the absence of a primer strand) protrudes from the thumb domain. This loop serves as a platform for the initiation of the polymerization activity. In the absence of RNA, the thumb is positioned near the fingers subdomain and interacts with the priming loop. Several loops (named “the fingertips”) connect the fingers and thumb subdomains. These flexible loops are proposed to play an important role in controlling the conformational changes during the RdRp activity [40]. The available structures of the full-length NS5 and of the RdRp domain were obtained without bound RNA, and both display a closed conformation with a RNA binding tunnel too narrow to accommodate a dsRNA substrate [40,45,46]. Therefore, the structure is thought to depict a preinitiation state. It is not clear whether the transition to an open conformation would occur directly after the synthesis of a priming dinucleotide or a tri-nucleotide or whether this event requires formation of a longer primer (see below section on RdRp activity). Nonetheless, an outwards rotation of the fingers subdomain relative to the palm subdomain and a retraction of the priming loop is expected, when the enzyme transitions from the initiation state towards the dsRNA elongation state. These concerted movements would result in an increase of the volume of the RNA tunnel and enable the translocation of the nascent dsRNA, as was finally observed in the case of the related HCV virus NS5B polymerase, following many years of studies [47]. These conformational changes are thought to be controlled by amino-acid motifs located in the connections between the fingers and the thumb subdomains. Unfortunately, in the crystal structures reported so far, these motifs are not visible due to their high flexibility in the absence of an RNA substrate. A comparison of the available structures of NS5 proteins from various viruses belonging to the *Flavivirus* genus (DENV3, JEV, ZIKV) consistently shows a closed conformation of the RdRP domain. However, a major difference resides in the various relative orientations between the RdRp and the MTase domains, leading essentially to two sets of interfaces, with the NS5FL from DENV being in one group with the NS5FL from JEV and ZIKV belonging to another related evolutionary group.

### 3.2. RdRp Activity

Flavivirus RdRps use RNA as a template and do not require a primer to elongate nascent RNA (so-called “de novo” activity). The RNA synthesis mechanism was proposed based on structures of RdRp from various viruses [18,19,41,47,48,49,50,51,52,53], in complex with single-stranded (ss)RNA or dsRNA, or in the presence of inhibitors. In the case of flaviviruses, the viral RNA secondary structures present at the 3’ and 5’ UTR of the genome along with its circularization play a crucial role in the replication activity of NS5 [53]. Upon binding the 3’ end of the RNA template, two bases, an ATP and a GTP are positioned through Watson–Crick interactions with the C and U bases located at the 3’ end of the template RNA. At this stage, the protein is believed to adopt a closed conformation: the priming loop is extended and blocks the exit of the RNA tunnel, and provides a stabilizing platform for the formation of a ribose-phosphate bond by a nucleophilic attack of the activated alcohol group of the adenine ribose on the guanine α-phosphate, producing the initial dinucleotide primer. The phosphate groups from A1 and G2, and subsequently between the 5’ end of the newly synthetized strand and the incoming nucleotide, are positioned through interactions with the metal ion bound by aspartic acid 665 and 666. This conformation favors the nucleophilic attack of the activated 2’-*O*-ribose on the α-phosphate of the incorporated nucleotide. The first published structure of the RdRp domain in complex with a chain terminator nucleoside analogue 3’dGTP mimicking the positioning of the first base of the newly synthetized RNA showed the importance of residues R729 and R737 in coordinating the phosphate groups. Moreover, a role for the indole group of W795 from the priming loop for stacking the base of the nucleotide was proposed, to position the first bases for efficient polymerization [40]. This was however challenged by our unpublished observations that mutagenesis of W795 did not affect initiation [54] and later a convincing case for the role of H798 for this task was put forward by the group of Bruno Canard [55].

Once the first ribose-phosphate bond is formed with the production of pyrophosphate as a by-product, the dsRNA is then translocated into the exit tunnel to allow the positioning of the next nucleotide. Thus, the mechanism of RdRp can be broken down into four steps: (1) the protein is in a resting mode in a preinitiation form, most probably in the closed conformation seen in the crystal structures with the priming loop in an extended conformation; (2) in the initiation state, the 3’ end of the ssRNA template is bound along with the A and G ribonucleotides until the initial phosphodiester bond is formed. Here the priming loop acts as a stabilizing platform maintaining the adenosine in position to allow synthesis of the initial dinucleotide primer; and (3) the NS5 protein undergoes a large conformational change with a concomitant retraction of the priming loop leading to an opening of the dsRNA exit tunnel. This conformational change is thought to be the rate-limiting step for the RNA polymerase activity [50]. Finally, the protein is locked in an open conformation, allowing processive RNA polymerization.

Until recently, NS5 was thought to solely use RNA as a template. In the case of the Zika virus, a recent study suggested that ab-initio polymerization is also possible using ssDNA as template. Moreover, the elongation reaction can occur with a heteroduplex formed by a DNA template and an RNA primer [56]. Interestingly this feature is present in the DENV NS5 full-length protein whose affinity for dsDNA is similar to ssRNA, as shown by fluorescence studies [57]. These findings show that the RNA binding tunnel can accommodate two types of nucleic acids revealing a certain plasticity. In vitro, the DENV NS5 RdRp can use either Mg^2+^ or Mn^2+^, whilst ZIKV NS5 can only use Mn^2+^ and is inhibited by Mg^2+^. However, the in vivo metal dependency of the enzymatic activity is not clear yet. 

Interestingly, the effect of the presence of the MTase domain stimulates the RdRp activity; moreover, in the presence of residues from the linker region, both the activity and stability of the RdRp domain is significantly enhanced [36,58,59]. The MTase domain affects both the initiation and elongation steps. Therefore, the two domains “communicate” through the interface between them which contains a series of evolutionary-conserved residues [60]. In the case of NS5 from JEV, changing the conserved residues at the interface affects the initiation and the elongation in different ways [61]. A key element in the genomic RNA also affecting the NS5 activity is the secondary structures adopted by a specific region called “stem-loop A” or SLA within the the 3’UTR [62]. The SLA region is responsible not only for the recruitment of the NS5 protein through direct interactions but also for the initiation of the negative strand polymerization activity [63]. This interaction is also mapped to the F1 motif of the NS5 RdRp [44] and more specifically to the two lysine residues 456 and 455, whose mutations abolish activity, although binding to the template RNA remains possible. 

## 4. Linker Domain and Protein Flexibility

Detailed information on the cooperation between NS5 and NS3 proteins and how the helicase and RdRp activities and the triphosphatase and capping activities are subjected to a cross-talk between the various domains is still lacking. Thus, it is not clear how a newly synthetized RNA strand is separated from the template, undergoes removal of the 5’ pyrophosphate, and becomes guanylylated before methylation. The various enzyme activities are handled by different proteins and the cognate domains would need drastic conformation changes that are not easily visualized based on the available crystal structures of the NS3 and NS5 protein, and on what we know from their interaction. We cannot exclude the possibility that synthesis and capping are performed in *cis* in a sequential manner but it is easier to conceive that several molecules cooperate in *trans*, each one adding a modification to the RNA substrate. Comparison of the overall structures of DENV, ZIKV and JEV NS5 proteins shows that although the folding of the distinct domains is conserved (root mean square deviation (rmsd) ~1Å for the MTase and the RdRp domains respectively, see PDB access codes 4V0R, 5TFR and 4K6M), their relative orientation changes by a rotation of ≈100°. The linker domain has been proposed to allow the MTase and the RdRp domains to adopt different relative conformations upon binding to RNA, NS3 or host partners. The linker region of NS5 (residues 266–271) connects the MTase and the RdRp domains. In the crystal structures of the full length DENV3 NS5; the linker comprises a short 3_10_ helix of four residues [58]. This region determines the relative orientation of the two domains and allows the protein to adopt a range of conformations in solution. Moreover, it affects the activity of the RdRp domain [42]. The linker region residues are also involved in the interface between the two domains. In the case of JEV NS5 protein, the linker is not visible in the crystal structure, the two domains are in a different relative orientation compared to the protein from DENV. Nonetheless it was shown that the interaction of the MTase and the RdRp domains also affects the replication activity [64]. The linker residues increase the stability of the isolated RdRp domain and affect the stability of the proteins in the different serotypes [65]. 

**Figure 4:**
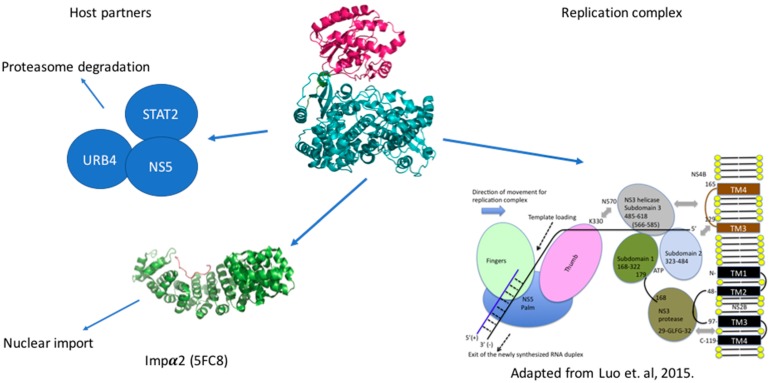
DENV NS5 interactome. Schematic view of the interactions established by NS5 in the infected cells. In the endoplasmic reticulum (ER) vesicles NS5 interacts with NS3 to replicate and cap the genomic RNA. NS5 brings the STAT2 protein and the URB4 close and triggers the degradation of the formed complex. The C-terminal region is recognized by the Impα2 protein for the import of NS5 protein into the nucleus.

NS5 protein has been considered to be active as a monomer. A recent study reported a crystal structure of the full length NS5 also from DENV [66], where two molecules were present in the asymmetric unit in a dimeric conformation. However, in solution, small-angle X-ray scattering (SAXS) studies have found no evidence for such a dimer formation [66]. NS5 protein in the replication complex is proposed to be in the vicinity of NS3 protein as it unwinds the dsRNA, allowing NS5 to use the ssRNA as a substrate [67], and NS5 protein has been proposed to enhance NS3 triphosphatase activity [33,34,36]. Residues from the region called αNLS (previously thought to be the nuclear localization signal; see below) have been reported to mediate the interaction with NS3 protein [36]. Overall, there is currently no high-resolution view about the macromolecular organization in the replication complex. Molecular interactions were described between NS3 and NS5 and between NS3 and NS4B, accounting for the anchoring of the replication complex in the ER membranes, because of the integral membrane association of NS4B and NS2B (Figure 4). However, we still lack information about the spatial organization allowing a coupling of dsRNA unwinding, polymerization and capping of the newly-synthetized RNA, keeping in mind that NS3 holds the protease activity, the helicase activity, the triphosphatase activity and that the latter is essential for the guanylyl transferase activity of the MTase domain.

## 5. Host Interacting Partners

In addition to its central role in genome replication, NS5 proteins from flaviviruses play a crucial part in evading the host immune system. Amino acids between residues 202 and 306 from the N-terminal region of the protein from the dengue 2 serotype have been shown to interact directly with STAT2 (Figure 4) [21,68,69,70] and promote its proteasome-assisted degradation in order to block type I interferon immune response, through the prevention of STAT1/2 complex formation. In cells expressing only NS5, the immune response is dampened but without an effect on the level of STAT2. It is only when the entire replication complex is expressed (for instance in the replicon system) or in the presence of the cleaved N-terminal region of NS5 between NS4B and NS5, that degradation is observed [21,71]. This involves the interaction of NS5 with STAT2 and ubiquitin ligase E3 recognin 4 (UBR4) simultaneously leading to the ubiquitination of STAT2 and its proteosomal degradation of [21]. The faith of NS5 however is not clear as it has not been excluded that NS5 would also be degraded in this process [71]. In the case of ZIKV NS5, the STAT2 degradation is observed even in absence of such a cleavage and seems to be independent of UBR4. The WNV NS5 protein is also able to bind to prolidase [72], a peptidase that promotes cell surface expression of the IFN-I receptor. This interaction prevents the maturation of the receptor, and thus its proper insertion in the plasma membrane and the transduction of the IFN-I signaling pathway, inhibiting the normal activation of the primary immune defenses upon viral infection. This shows the capacity of flaviviruses to inhibit the interferon immune response at various nodes along the signaling pathway [71].

Surprisingly, in infected cells, NS5 protein is mainly localized in the nucleus rather than in ER vesicles, especially in the DENV 2, 3, 4 serotypes [73,74]. This is linked to a decrease of interleukin (IL)-8 level but to an increase of chemokine production [75]. However, no link between the propensity of NS5 to be localized in the nucleus and viral fitness has been found nor how this nuclear localization can help the virus. Initially, this prominent localization in the nucleus was thought to be due to the presence of a nuclear localization signal in the interdomain region of NS5 protein. However, a recently reported crystal structure of full-length NS5 protein [76,77] showed that the proposed region is not easily accessible to the importin α/β responsible for translocation into the nucleus. Very recently, the 18 C-terminal residues of NS5 were proven to be essential for the import of NS5 protein into the nucleus (Figure 4) [78]. Moreover, the crystal structure of the complex between importin α and a peptide consisting of the C terminal 18 residues could account for the affinity differences observed between NS5 from the various DENV serotypes and the NLS. These affinities were in line with the variations observed in the NS5 localization between the four DENV serotypes [73]. Identification of putative nuclear partners and the validation of such interaction is essential to understanding NS5 activity in the nucleus. Dengue infection in HEK-293 cells up-regulates the expression of 21 genes [75]. When NS5 protein is expressed alone, Regulated on Activation, Normal T Cell Expressed and Secreted (RANTES) expression is up-regulated by nuclear factor-κB (NF-κB). The binding of the latter to the promoter of RANTES is enhanced in the presence of NS5 [75]. Another study also showed that tumor necrosis factor α (TNF-α) expression was also up-regulated by NF-κB in presence of NS5 protein. The direct binding of NS5 to NF-κB has not been shown, but NS5 was proposed to bind to the Daxx protein [79], and compete with its interaction with NF-κB. This would lead to NF-κB release and its interaction with the RANTES promoter and the up-regulation of its expression. RANTES is a chemokine, involved in the recruitment of immune cells to the inflammatory site. NS5 protein was also found to be Small Ubiquitin-like Modifier (SUMO)ylated both in vivo and in vitro and a SUMOylation site was identified in the N-terminal domain of the protein. This posttranslational modification seems to increase the stability of the protein and its reduction yields to less effective replication of DENV. Interestingly, SUMOylation of NS5 is required for NS5 mediated suppression of IFN response [70].

## 6. Antiviral Strategies against NS5

A comprehensive review of the antiviral efforts directed against DENV was recently published [80]. Inhibiting RNA polymerization is a prime target for treating viral infections in general [16,45]. This strategy was successfully used for the treatment of HCV infections by targeting the homologous NS5B protein [16]. NS5, being the most conserved protein, is therefore a target of choice for designing a pan-serotype antiviral compound. As seen above, two essential activities for viral replication are harbored by NS5: RNA capping, and polymerization. Both have been targeted and several compounds have been developed that interfere with these activities. The molecules targeting NS5 can be divided into two classes: the nucleoside inhibitors (NIs) and the non-nucleoside inhibitors (NNIs). The first class mimics the natural substrates of the enzyme and requires phosphorylation by host kinases. NIs are administrated as a pro-drugs, while the second class is not. The discovery of compounds is highly dependent on the development of reliable inhibition assays amenable to high throughput screenings (HTS) using various compound libraries. The selection of lead molecules with NS5 inhibitory activity employed X-ray crystallography (to identify fragments that bind to the protein [81]), or were based on enzymatic inhibition assay or virtual screenings [82,83]. Both the initiation step and the elongation step [84,85] or capping activity were targeted [86]. However, compared to HCV, development of flavivirus antivirals has been much slower due to a variety of economic and biological factors. DENV and ZIKV have tropism for the brain, the testes and the lymph nodes [87]. Nucleoside inhibitors (NIs) administrated as prodrugs, which become activated inside the cell, will mimic natural NTPs preventing RNA polymerization or leading to a corrupted product. This strategy was successfully used against several viruses like HBV or HCV and in the case of HCV has led to the development of the remarkably potent HCV drug sofosbuvir (Table 1). NIs present the great advantages of targeting active sites, thus they are used in a pan-serotype or even a cross-reactive strategy and reduce the probability of resistance. However, NI side effects are difficult to predict in vitro because they also interfere with several cell processes. Several promising NIs targeting dengue virus NS5 and other flaviviruses have failed in preclinical and clinical trials due to severe toxicity issues (Table 1). This is probably due to the tropism of the flavivirus present in compartments of the body that are not easily accessible for such drugs. Interestingly, sofosbuvir, the molecule used to treat chronic HCV infections, is active against ZIKV in replicon cell lines and prevents the death of mice infected with the virus [88,89]. A similar repurposing strategy was used to develop a potent anti-DENV drug starting from HCV inhibitors. However, in all published cases reviewed in [90], despite tedious medicinal chemistry efforts towards improving specificity and pharmacokinetic properties, the molecules either lose potency or produce toxic products due to interactions with host proteins (Table 1). To design an efficient NI against DENV or ZIKV, the structure of the ternary complex of the enzyme with the template RNA and the elongated primer would reveal important information. Such a structure will help the design of a specific and potent compound specific, while minimizing off-target interactions.

The second class of compounds are the non-nucleoside inhibitors (NNIs, Table 1). They are non-competitive inhibitors that bind to specific pockets other than the active site [81]. They act by blocking conformational changes of the enzyme, typically between the initiation and the elongation steps or hindering the binding of the RNA. Their biggest advantage is a high specificity for their allosteric binding pocket, drastically minimizing off-target effects. However, this is also a weakness as these pockets are more prone to mutations than the active site, leading to the emergence of resistant variants. Moreover, these compounds might have poor pharmacokinetic properties limiting their bioavailability. As described above, RNA replication by the polymerase activity can be broken up in the initiation step where the first two/three nucleotides are linked and an elongation state where this “primer” is used to elongate the RNA strand. Between these steps, a conformational change is thought to occur in order to accommodate the dsRNA. Thus, in principle either the initiation, the elongation and intermediate states in-between can be targeted. The strategy to develop NNI starts with a HTS screening based on structural determination of complexes with fragments originating from the library of compounds to identify potential molecules. This is followed by rounds of binding biophysical characterization and chemical optimization, using medicinal chemistry principles. Until recently no allosteric pocket for NNI binding had been described for dengue virus RdRp although compounds targeting domains outside of the active site (mostly thumb site inhibitors) had been reported. The recently described “N pocket” [94] is localized near the active site, at the junction of the thumb and the palm subdomains in DENV serotype 3. A crystallographic compound screening allowed the identification of this pocket. Then structure guided modifications of the initial hit led to molecules potently inhibiting polymerase activity (Table 1). Study of the properties and the inhibition mechanism showed that the compound inhibited the initiation step of the RNA polymerization in all DENV serotypes. Although the compound cannot be used for in vivo studies because of it relatively poor pharmacokinetics properties, this work clearly identified an allosteric pocket from DENV that is essentially shared by the ZIKV RdRp and that can now be targeted using rational design. Another interesting NNI compound was also identified by HTS based on an activity assay and on optimisation of identified hits [97,98]. UV-cross-linking of the compound showed that it binds the RdRp domain at the edge of the RNA tunnel blocking the template access and interfering in the transition between the open and close conformations of the protein. In vitro the compound showed promising inhibition activity but its poor pharmacokinetic properties did not permit the development of this compound.

Methylation of the RNA cap is essential for virus replication and represents an important drug target [26,27,28,99]. In the case of West Nile virus, defects in MTase can lead to weakened [100] or abolished [101] viral replication capping activity. The capping activity can be inhibited either by targeting the guanylyl transferase activity, the N7 or the 2’O methylation activities. Targeting the SAM pocket is an established antimicrobial strategy and several analogues to this ubiquitous molecules exist such as sinefungin [16], a broad spectrum MTase inhibitor having anti-parasitic activity. This inhibitor has been shown to bind to the SAM pocket of several flaviviruses. Interestingly, its affinity is higher than SAM. However, this compound cannot be used as a drug due to its lack of specificity that will provoke severe side effects since it is not specific to the viral MTase. Thus, the SAM scaffold was modified by adding substituents at the level of the adenine ring. The structure of the 2’O methylation ternary complex could also provide inspiration for the design of novel inhibitors, particularly with a recent study showing the guanylyl transferase reaction [24]. Compounds binding to the GTP pocket might inhibit all three activities by blocking the access of the GTP (for the guanylyl transferase activity, the methylation of the guanine N7 and the methylation of the ribose 2’O). HTS binding assays identified compounds binding to this pocket and inhibited virus replication, thus validating the strategy. A recent study also showed that a compound can efficiently and specifically inhibit the viral MTase with few cytotoxic effects [82]. For this, a virtual screening is performed to select possible binders that would later be tested in vitro to assess activity and absence of cytotoxicity. The binding is also characterized by obtaining the crystal structure of the complex with the protein, allowing eventual optimization of the compound [82]. The main advantage of this recently identified molecule is its interactions with residues outside of the SAM binding pocket, increasing the specificity towards the pathogen’s MTase. An interesting strategy was recently used to link compounds obtained through fragment based docking that have been shown to be active as standalone molecules targeting the MTase domain, to improve their potency and specificity [96]. However, although biochemical activity is improved for the linked compound compared to the initial hits, no activity was observed in the in vitro assays, probably due to poor membrane crossing of the compounds.

Recently, an alternative approach to design NNIs targeting disordered regions of NS5 has been proposed [102]. The idea is to target the structural plasticity of NS5 to affect its activities. However, details on the disordered regions must be obtained to aim specifically and efficiently affect the protein activity. A challenging alternative for the identification of new specific compounds is to target the interaction network within the replication complex and with the host cell protein partners, with a view to disrupt key protein–protein interactions [103,104,105]. An obvious target for such a strategy is to target the regions of interaction between the elements of the replication complex (NS3-NS5, NS3-NS4 for example). Assays to disrupt the interaction between NS3 and NS5 proteins are being developed [106]. Another interesting approach would be to disrupt the interaction between with identified partners like Daxx or STAT2. This type of molecules would be very interesting if used in complement of an activity inhibitor of NS5 (NIs or NNIs) to enhance their potential. However, to screen for such compounds, robust assays need to be developed. The intrinsically disordered regions might in this case play an important role [102]. Another example is with respect to the NS5 and NS3 proteins of JEV that interact with translation elongation factor 1A1 [107]. This interaction enhances the viral replication. However, a microRNA (miRNA; miRNA-33a-5p) targeting the expression of elongation factor 1-α1 (EF1A1) can modulate the fitness of JEV in infected cells. JEV infection down-regulates the expression of miRNA-33a-5p to limit its effects. This example where expression of the host protein is aimed to affect virus replication is a good illustration of an antiviral strategy that would target critical host partners of viral proteins rather than the virus directly. Identifying the exact partners and characterizing their interactions with the replication would shed light on new antiviral targets to treat dengue infections.

## Figures and Tables

**Table 1:** Examples of NS5 inhibitors. NIs and NNIs targeting RdRp and MTase activity with half maximal inhibitory concentration (IC50) values and in vitro or in vivo characteristics. NNIs: non-nucleoside inhibitors; NIs: nucleoside inhibitors.

Molecule		Type of Inhibitor	Target Activity	IC50 (μM)	Characteristic	Reference
Sofosbuvir	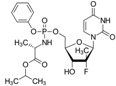	NI	RdRp		Not active against DENV	[91]
NITD 008	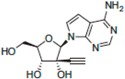	NI	RdRp	0.7	High cytotoxicity	[92]
NITD 203	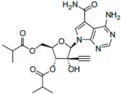	NI	RdRp	0.1–0.7	High cytotoxicity	[93]
N-pocket Compound 27	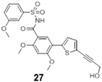	NNI	RdRp	3.9	Toxic in animal model	[81,94,95]
N-pocket Compound 29	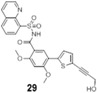	NNI	RdRp	1.9	Toxic in animal model	[81,94,95]
Entry 30	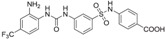	NNI	MTase	91 (DENV2)	Poor inhibition potency	[96]
Compound 10	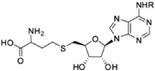	NNI	MTase	0.08–0.24 µM (DENV3)	Poor inhibition potency	[29]
NSC 306711	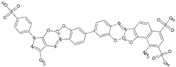	NNI	MTase	1 µM	Candidate for optimization	[82]
